# Role of Omentin, Vaspin, Cardiotrophin-1, TWEAK and NOV/CCN3 in Obesity and Diabetes Development

**DOI:** 10.3390/ijms18081770

**Published:** 2017-08-15

**Authors:** Xavier Escoté, Saioa Gómez-Zorita, Miguel López-Yoldi, Iñaki Milton-Laskibar, Alfredo Fernández-Quintela, J. Alfredo Martínez, María J. Moreno-Aliaga, María P. Portillo

**Affiliations:** 1 Department of Nutrition, Food Sciences and Physiology, University of Navarra, 31008 Pamplona, Spain; xescote@unav.es (X.E.); mlyoldi@alumni.unav.es (M.L.-Y.); jalfmtz@unav.es (J.A.M.); mjmoreno@unav.es (M.J.M.-A.); 2 Centre for Nutrition Research, University of Navarra, 31008 Pamplona, Spain; 3 Nutrition and Obesity Group, Department of Nutrition and Food Science, University of the Basque Country (UPV/EHU) and Lucio Lascaray Research Institute, 01006 Vitoria, Spain; saioa.gomez@ehu.es (S.G.-Z.); inaki.milton@ehu.eus (I.M.-L.); mariapuy.portillo@ehu.es (M.P.P.); 4 Spanish Biomedical Research Centre in Physiopathology of Obesity and Nutrition (CIBERobn), Institute of Health Carlos III, 01006 Vitoria, Spain; 5 Navarra Institute for Health Research (IdiSNa), 31008 Pamplona, Spain

**Keywords:** adipokines, omentin, vaspin, cardiotrophin-1, TWEAK and NOV/CCN3

## Abstract

Adipose tissue releases bioactive mediators called adipokines. This review focuses on the effects of omentin, vaspin, cardiotrophin-1, Tumor necrosis factor-like Weak Inducer of Apoptosis (TWEAK) and nephroblastoma overexpressed (NOV/CCN3) on obesity and diabetes. Omentin is produced by the stromal-vascular fraction of visceral adipose tissue. Obesity reduces omentin serum concentrations and adipose tissue secretion in adults and adolescents. This adipokine regulates insulin sensitivity, but its clinical relevance has to be confirmed. Vaspin is produced by visceral and subcutaneous adipose tissues. Vaspin levels are higher in obese subjects, as well as in subjects showing insulin resistance or type 2 diabetes. Cardiotrophin-1 is an adipokine with a similar structure as cytokines from interleukin-6 family. There is some controversy regarding the regulation of cardiotrophin-1 levels in obese -subjects, but gene expression levels of cardiotrophin-1 are down-regulated in white adipose tissue from diet-induced obese mice. It also shows anti-obesity and hypoglycemic properties. TWEAK is a potential regulator of the low-grade chronic inflammation characteristic of obesity. TWEAK levels seem not to be directly related to adiposity, and metabolic factors play a critical role in its regulation. Finally, a strong correlation has been found between plasma NOV/CCN3 concentration and fat mass. This adipokine improves insulin actions.

## 1. Introduction

Adipose tissue was originally considered to be a passive reservoir for energy storage, mechanical and heat insulation, and to participate in the regulation of thermogenesis [1]. Nowadays adipose tissue is regarded as an active endocrine organ which, in addition to regulating fat mass and nutrient homeostasis, also releases a large number of bioactive mediators called adipokines. In fact, over recent decades, adipose tissue has turned out to be an extraordinarily active endocrine organ [2,3]. The secretion of adipokines seems to be depot specific, with some of them primarily secreted in visceral adipose tissue and others in subcutaneous adipose tissue [3].

Adipokines influence the adipocyte function in an autocrinic or paracrinic manner and also affect multiple metabolic processes endocrinically [4]. Adipokines participate in the regulation of multiple biological processes, such as feeding behavior, insulin sensitivity, inflammation, angiogenesis, blood pressure, fibrinolisis, immunity, and vascular homeostasis.

The present review focuses on several of the recently discovered adipokines, omentin-1, vaspin, cardiotrophin-1, TWEAK and NOV/CCN3. More specifically, it focuses on their relationship with obesity, insulin resistance and diabetes. In the case of omentin-1 and vaspin, the review mainly describes those studies carried out in humans. As far as rodent studies are concerned, those describing mechanisms of action have also been included in order to better characterize the actions of these adipokines. In the case on the other three adipokines, due to the small number of studies addressed in humans, those performed in rodents have also been described.

## 2. Omentin

Omentin is a 38–40 kDa adipokine which was identified from a cDNA library in visceral omental adipose tissue by Yang et al., in 2003 [5]. Adipose tissue is mainly composed by mature adipocytes, but there is also a mixed population of cells known as the stromal-vascular fraction, which includes: preadipocytes, macrophages, lymphocytes, endothelial cells [6]. Omentin is predominantly produced by the stromal-vascular fraction of visceral adipose tissue, but not by mature adipocytes [7]. It is not known so far which of these cell types expresses omentin. The production of omentin by subcutaneous adipose tissue is extremely low [7]. Thus, omentin is among the first molecules known to exhibit such a dramatic difference in gene expression between the two major fat depots. In addition, other cells, unrelated to adipose tissue, such as intestinal Paneth cells [8], can also contribute to omentin production in the body. Moreover, omentin is scarcely expressed in mouse visceral adipose tissue. This finding suggests that in all likelyhood this adipokine plays a more important role in humans than in mice [7].

There are two omentin genes, located adjacent to each other in the 1q22–q23 chromosomal region, which produce omentin-1 and omentin-2 [9]. Both isoforms show different pattern of tissue expression. In humans, omentin-1 is the predominant isoform in plasma and adipose tissue [10]. The administration of glucose and insulin to human omental adipose tissue explants results in a dose-dependent reduction of omentin-1 expression. Furthermore, prolonged insulin-glucose infusion in healthy individuals induces a significantly decreased plasma omentin-1 concentration [5]. These results suggest that omentin-1production is under glucose and insulin regulation. 

Physiological concentrations of omentin-1 in humans are in the range of 100–800 ng/mL [11]. It is known that its expression and production are modified in several pathological situations, such as obesity and insulin resistance. Omentin-1 expression is also altered in inflammatory states [12]. Thus, taking into account that obesity itself is associated with low levels of chronic inflammation, this may contribute to the increase in omentin-1 concentration observed in obese subjects.

### 2.1. Omentin and Obesity

With regard to obesity, De Souza Batista et al. (2007) reported that serum omentin-1 concentration, as well as visceral adipose tissue omentin expression, were significantly lower in overweight and obese subjects than in lean ones [13]. In this study, a sex difference in circulating omentin-1 levels was also observed when comparing all women with all men, adjusted for body mass index (BMI), and when comparing lean women with lean men. Women showed higher concentration of circulating omentin-1 than men. This is in line with the study reported by Tan et al. where the expression of mRNA for omentin-1 in adipose tissue and omentin-1 serum concentration correlated negatively with 17β-estradiol [14]. Moreover, in such study significant negative correlations, based on variance components analysis adjusted for sex, age, and family structure, were found between plasma omentin-1 concentration and BMI, waist circumference and homeostatic model of assessment for insulin resistance (HOMA-IR). The authors emphasized that circulating total serum adiponectin concentrations were positively correlated with plasma omentin-1 values. They proposed that an inverse relationship between obesity and both omentin-1 and adiponectin may suggest similar regulation. 

Similarly, Auguet et al. (2011) reported significantly lower serum omentin-1 level in morbidly obese women than in normal weight women, and that this concentration inversely correlated with glucose metabolism markers. Moreover, omentin expression in visceral adipose tissue was significantly lower in the morbidly obese women [15]. Also, in morbidly obese subjects, Cătoi et al. (2014) found that omentin-1 levels were decreased when compared with normal weight healthy subjects, and that they were inversely associated with chronic inflammation. Finally, they reported that two of the main modulating factors seemed to be hyperglycemia and BMI [16].

The relationship found between omentin-1 and obesity has also been found in adolescents and children. Oświęcimska et al. (2015) measured plasma omentin-1 concentration in lean and obese adolescent girls (1516 years old) not showing metabolic alterations. They observed that it was significantly lower in obese than in normal weight girls. The differences were still present after adjustment for BMI [17]. In the case of children, Catli et al. (2013) reported lower serum omentin-1 concentration in obese children. Moreover, this parameter was negatively correlated with BMI, waist circumference, HOMA-IR and insulin levels, suggesting that omentin-1 might be a biomarker for metabolic dysfunction also in childhood [18]. Nevertheless, there are also a study showing that higher circulating omentin-1 was associated with a less favorable metabolic profile, which includes insulin resistance, markers of adipose tissue metabolism, blood pressure and family history of metabolic dysfunction, in asymptomatic pre-pubertal children (7 years old). The authors concluded that this fact can represent a compensatory mechanism whereby omentin-1 attenuates these metabolic abnormalities [19].

Taken together these results clearly show that obesity reduces serum omentin-1 concentration and adipose tissue secretion in adults and adolescents. In the case of children, the reduced number of reported studies and the discrepancies between them indicate that further studies are needed to be sure if the same is true in this population. 

Conversely, body weight reduction induces increased omentin-1 serum concentrations. As far as energy restriction, the main tool used in obesity treatment, is concerned, Moreno-Navarrete et al. (2010) reported a significant increase in serum omentin-1 concentration in obese subjects submitted to a daily energy deficit of 500–1000 kcal/day for 4 months [20]. With regard to potential sexual dimorphism, the results reported by these authors were not in good accordance with those published by De Souza Batista et al. (2007) [13] in overweight and obese subjects, because omentin-1 concentration, measured by ELISA, was higher in men than in women, although the increase induced after weight loss was similar in both groups. It is important to point out that in the study reported by De Souza Batista et al. [13] omentin was measured by western blot. Thus, the discrepancy between both studies could be due, at least in part, to the different analytical approach.

In the study reported by Urbanová et al. (2014) a stronger energy restriction was used as a therapeutic strategy. Morbidly obese women, showing type 2 diabetes or not, underwent a 2-week very low calorie diet (VLCD) with 600 kcal/day intake. Surprisingly, this dietary strategy had no significant effect on serum omentin-1 concentrations in any group studied. Although the main depot involved in omentin production is visceral adipose tissue, the authors measured mRNA expression of omentin-1 in subcutaneous adipose tissue and observed that it was not affected by VLCD in any group studied. The researchers considered that the lack of effect could be due to the short-term dietary intervention [21].

Another important strategy used in the treatment of obesity and glucose homeostasis alterations (insulin resistance and diabetes) is to increase physical activity. In this context, Saremi et al. (2010) observed that, at baseline, normal weight participants had significantly higher serum omentin-1 concentrations than did overweight and obese participants, and there were inverse correlations between omentin-1 and waist circumference, fasting glucose, and insulin resistance. After the aerobic training, waist circumference, percentage of body fat, fasting glucose and insulin resistance significantly decreased in overweight and obese subjects, while serum omentin-1 concentration significantly increased [22]. These findings suggest that exercise-induced changes in omentin-1 may be associated with the beneficial effects of exercise on insulin sensitivity and weight reduction.

Other authors have also reported data concerning the effects of exercise. Thus, Aminilari et al. (2017) addressed a study to compare the impact of 12 weeks of aerobic exercise, resistance exercise or a combined program of aerobic and resistance activity on HOMA-IR, serum omentin-1 concentration and in body fat, in middle-age diabetic women [23]. Fasting blood sugar decreased significantly in all intervention groups, while HOMA-IR decreased only in the aerobic and combined exercises groups. Furthermore, there was a significant increase in the omentin-1 level only in the combined exercise group. Similar results have been found by other authors in children. Thus, after 16 weeks of an exercise training program, obese children showed weight loss, which was accompanied by decreases in BMI, waist circumference, body fat percentage, insulin, and HOMA2-IR, although the subjects remained obese because of the short intervention. Furthermore, serum omentin-1 levels were also significantly increased. Negative significant correlations between omentin-1 changes and changes in BMI, waist circumference, body fat percent, fasting serum insulin levels and HOMA2-IR were also found. Based on the results of multiple linear regression analyses, the authors revealed that changes of BMI and insulin levels were independent predictors of the difference observed in serum omentin-1 levels after an exercise program [24].

However, in other studies changes in omentin-1 plasma levels from overweight women (aged 25–45 years) after an exercise program, consisting of rhythmic aerobic exercise (55–85% maximum heart rate) along with core stability training, were not found [25]. As stated by the authors, a more intense training seems to be required to significantly modify plasma omentin-1 levels.

The conclusion of these studies is that the strategies used to induce a reduction in body weight, such as dietary energy restriction and physical activity, are effective in correcting the alterations induced in omentin-1 serum concentration induced by overweight and obesity.

### 2.2. Omentin and Alterations in Glycemic Control

As far as alterations in glycemic control are concerned, several authors have documented lower levels of serum omentin-1 in nascent metabolic syndrome; even after correction for obesity [26]. Shang et al. (2011) also reported lower serum omentin-1 concentration in patients with metabolic syndrome, however they did not correct data for the increased BMI and waist circumference, and thus they were unable to conclude that this correlation was a feature of metabolic syndrome per se [27].

Vu et al. (2014) carried out a study to determine the relationship between circulating omentin-1 concentration and components of the metabolic syndrome in adults without type 2 diabetes. They also evaluated whether sexual dimorphism existed for such relationships. Unlike other studies, no differences between subjects with or without metabolic syndrome were found [28]. The authors considered that this discrepancy with other studies was likely to be due to differences in study designs (e.g., inclusion and exclusion criteria, percentage of men and women, and age of subjects). Moreover, differences in terms of subject medication also existed among these studies. With regard to sexual dimorphism, no significant differences between men and women were observed when the entire study cohort was analyzed; however, men with metabolic syndrome had lower plasma omentin-1 concentrations than women did.

Moreover, studies in subjects showing type 2 diabetes have been addressed. Pan et al. (2010) carried out a study devoted to comparing serum omentin-1 levels in (a) subjects with normal glucose tolerance, (b) subjects with impaired glucose regulation, and (c) subjects with newly diagnosed and untreated type 2 diabetes [29]. Omentin-1 concentrations of fasting and 2 h post-glucose load were significantly decreased in diabetic subjects, as well as in subjects with impaired glucose regulation, when compared with the control group. Within each group, there was no difference in omentin-1 concentration before and after glucose load. Later, El-Mesallamy et al. (2011) observed significantly reduced serum omentin-1 concentration in type 2 diabetes patients, even after adjustment for the effect of some covariates such as age or BMI. Furthermore, simple linear analysis reflected that serum omentin-1 concentration was significantly negatively correlated with BMI, fasting blood glucose and HOMA [30]. Other authors also found lower omentin-1 concentrations in plasma in pre- or diabetic obese patients compared to normoglycemic subjects in a cross-sectional study [31].

Moreover, Wittenbecher et al. (2016) evaluated the longitudinal association of omentin-1 concentrations with the risk of type 2 diabetes. This observational study was based on 2500 randomly selected participants of the European Prospective Investigation into Cancer and Nutrition (EPIC)-Potsdam cohort. Despite inverse associations of omentin-1 with measurements of body fat and direct associations with adiponectin, the authors found no indication of a diabetes protective role of omentin-1 in prospective analyses. Unexpectedly, data suggested an elevated omentin-1–related diabetes risk among participants with high adiponectin concentrations [32]. 

The relationship between omentin-1 and diabetes is not limited to type 2 diabetes. Polkowska et al. (2016) observed the same situation in type 1 diabetes. Thus, serum omentin-1 concentration was significantly lower in children showing this type of diabetes than in control children, independently of the duration of this pathology [33].

### 2.3. Signaling Pathways Involved in the Metabolic Actions of Omentin

Given the above studies, scientific evidence clearly supports the role of omentin in obesity and glucose metabolism regulation. However, knowledge concerning the mechanisms involved are scarce so far. Brunetti et al. suggested that omentin could be involved in regulation of appetite. They observed that the central infusion of omentin-1 in Wistar rats (8 μg/kg body weight) did not show any acute effect on food intake, nor on the hypothalamic gene expression of agouti-related protein (AgRP), neuropeptide Y (NPY), orexin-A, amphetamine-regulated transcript (CART), corticotropin-releasing hormone (CRH) and pro-opiomelanocortin (POMC) [34]. By contrast, when omentin was administered intraperitoneally for 14 days it increased food intake (beginning on day 10) and body weight (beginning on day 12). Both in vivo and in vitro experiments showed a neuromodulatory role for omentin-1 on peptidergic and aminergic signaling. Omentin-1 reduces CART and CRH gene expression, in the hypothalamus, but no NPY, POMC, AgRP and orexin-A [35]. An increase in hypothalmic l-dopa and norepinephrine synthesis, without changes in dopamine and serotonine, was also observed [35].

With regard to glycemic control, Yang et al. (2006) observed insulin-stimulated glucose transport by increasing protein kinase b (Akt) phosphorylation (and thus activation) in human adipocytes, suggesting that omentin may improve insulin sensitivity [7]. The researchers also observed that omentin, enhanced only insulin-mediated glucose transport and did not stimulate basal glucose transport on its own, indicating that it has no intrinsic insulin-mimic activity. In addition, omentin increased the activity of insulin receptor substrate (IRS) due to the inhibition of mammalian target of rapamicin (mTOR-p70S6K), which in turn is a consequence of AMP protein-kinase (AMPK) activation [11].

**Figure 1:**
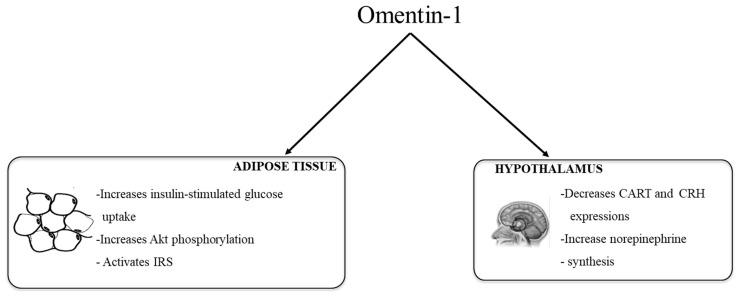
Schematic representation of the metabolic actions of omentin-1 in adipose tissue (data obtained from isolated human adipocytes) suggesting an improvement in insulin sensitivity and hypothalamus (data obtained from animal studies) suggesting increased food intake. Akt: protein kinase b, IRS: insulin receptor substrate, CART: amphetamine-regulated transcript, CRH: corticotropin-releasing hormone.

Figure 1 summarizes the main actions of omentin-1 in adipose tissue and hypothalamus.

## 3. Vaspin

Vaspin (visceral adipose tissue-derived serpin, serpinA12) is a member of the serine protease inhibitor family of serpins, recently proposed as a good-reliability biomarker, along with other pro-inflammatory adipokines, in evaluating exposure-disease associations [36]. Vaspin structure is made up by three β-sheets, nine α-helix and an exposed flexible reactive central loop (RCL) [37]. It was identified for the first time by Hida et al. in 2000 in the visceral adipose tissue of a rat model of obesity and type 2 diabetes (Otsuka Long-Evans Tokushima fatty rat; OLETF) [38]. The authors observed that the expression of this adipokine reached its highest values at the age when plasma insulin levels and obesity peaked. As the diabetes worsened, the expression of vaspin decreased [39]. 

Human vaspin protein is constituted by 414 amino acids (412 and 414 amino acids in rat and mouse vaspin, respectively) and has a 40% homology with α_1_-antitrypsin [39]. Vaspin is produced in humans by several tissues: adipose tissue, liver, skeletal muscle, pancreas and skin; the highest production is found in liver [40,41]. Unlike OLETF rats, vaspin expression was not restricted to visceral fat in humans, in fact, it was detectable in 23% of the visceral and in 15% of the subcutaneous adipose tissue samples in the study reported by Klöting et al. [42]. Moreover, in human adipose tissue, vaspin gene expression seemed to be differentially regulated in function of the fat depot [42]. Moreover, Lee et al. demonstrated that vaspin expression was greater in adipocytes than in the stromal vascular cell fraction of abdominal visceral adipose tissue [10]. Interestingly, these results are consistent with those previously found in OLETF rats [39].

Several studies have found higher vaspin concentrations in adult women compared with men [43,44,45,46,47], whereas others did not [48,49,50,51]. Based on the results obtained in omental tissue explants [52] some authors have proposed that visceral vaspin levels in women may be due to estrogens. However, in the study reported by Seeger et al. (2008) women were post-menopausal [44]. On the other hand, Xu et al., in their study devoted to analyzing the correlation between plasma vaspin and aging, reported that plasma vaspin increased with aging in both males and females but to a smaller extent in males [53]. However, the authors declared some limitations of the study including that they did not assess menopausal status, which might affect vaspin plasma concentrations [54]. Consequently, further studies are needed to know whether sexual dimorphism exists with regard to the production of vaspin. As far as younger populations are concerned, Körner et al. observed that vaspin increased with puberty in girls, but not in boys [40]. Ko et al. did not find gender differences in serum vaspin levels when pre-pubertal Korean children were studied [55]. This is in accordance with the study reported by Körner et al. which shows that gender differences arise during pubertal progression in girls and are not present in pre-pubertal children [40].

An interesting aspect is the circadian rhythm of vaspin. Thus, Jeong et al. studied 24-h profiles of circulating vaspin concentrations in relation to meal ingestion in healthy adults. Serum vaspin concentrations had a meal related diurnal variation, with a pre-prandial rise and a post-prandial fall. The diurnal pattern of serum vaspin concentrations was reciprocal to that of insulin and glucose, suggesting that the postprandial decrease in serum vaspin levels may be caused by energy intake itself or by increased insulin and/or glucose plasma concentrations [56]. Later on, Kovacs et al., demonstrated that the previously observed meal-related decreases in serum vaspin concentrations observed by Jeong et al. were indeed mediated by insulin, independently of nutrient intake and glucose concentrations [57].

The influence of physical activity on vaspin production has also been reported in the literature. Thus, Youn et al. conducted a study to analyze the effects of a training protocol (3 sessions of 60 min per week) on the circulating vaspin concentration in Caucasian subjects. They found greater serum vaspin levels after following the training protocol (when comparing with baseline values) in subjects with different glucose tolerance levels (normal, impaired and type 2 diabetes), and they concluded that elevated circulating vaspin during the first weeks of physical training could mediate the improvement of insulin resistance in response to exercise [43]. Subsequently, to further elucidate the putative effects of physical exercise on circulating vaspin in healthy young men, this research group analyzed serum concentrations in response to two different exercise interventions: (a) before and after 1 h of resistance circle training and (b) before and after a 4-week exercise intervention. The experimental groups used in this study were (a) athletes, (b) previously trained lean, (c) previously untrained lean subjects or (d) previously untrained obese subjects. The conclusion of this study was that increased oxidative stress following short-term and long-term physical training decreases vaspin serum concentration, whereas changes in insulin sensitivity do not seem to regulate circulating vaspin [58]. In view of these results they proposed that the increase in serum vaspin concentrations found in patients with type 2 diabetes, normal or impaired glucose tolerance, in their previous study [43] either suggested that patients had unrecognized antioxidant supplementation and thus increased oxidative stress induced by exercise did not take place, or that the training intensity in this previous study was not sufficient to chronically increase oxidative stress. 

### 3.1. Vaspin and Obesity

With regard to this issue, while several studies showed an association between vaspin and obesity [43,52,59,60,61], others did not find it [15,54]. 

Auguet et al. (2011) reported that serum vaspin levels were not increased in morbidly obese women and that they did not correlate with BMI and markers of glucose or lipid metabolism [15]. Similarly, Sperling et al. (2016) found no differences of vaspin concentrations depending on body weight and body fat in a study carried out with obese individuals categorized into two groups, differing in their normal or abnormal response to a glucose tolerance test [54].

In the study reported by Cho et al. (2010), the relationship among BMI, cardio/respiratory fitness (CRF) and serum vaspin levels was analyzed in young Korean men. The main conclusion of this study was that high body fatness and low CRF were associated with increased serum vaspin levels. The authors suggested that the vaspin increase could act as a compensatory mechanism against insulin resistance (as it was shown by increased fasting insulin and HOMA-IR) [59].

Furthermore, Youn et al. analyzed the influence of BMI and body fat on vaspin production in normal glucose tolerant Caucasian subjects. Serum vaspin concentration was lower in subjects showing normal weight than in subjects showing overweight; this value was in turn lower than that found in obese subjects. Moreover, the authors divided the subjects into two BMI matched groups (26 kg/m^2^) with different percentage of body fat (<20% or >20%). In this case, higher serum vaspin concentrations were found in the group of subjects with >20% body fat [43]. This association has also been found in subjects with metabolic syndrome. Tan et al. observed that serum vaspin concentration correlated positively with BMI, waist circumference and percentage of body fat, which all reflect the degree of obesity. Among these variables, the percentage of body fat was associated most strongly with vaspin concentration [52]. Further, the same positive association between serum vaspin concentrations and body fat mass was reported in a study involving obese and healthy volunteers [61]. Interestingly in the study reported by von Loeffelholz et al. an interaction between serum vaspin, sex and BMI was found in normal weight and lean subjects, while no such association was detected in individuals with a BMI ≥ 25 kg/m^2^ [45]. Chang et al. (2010) conducted a study devoted to analyzing the association between serum vaspin concentrations and abdominal adiposity. For this purpose, visceral adipose tissue depot (VAT) area and homeostatic model assessment of insulin resistance (HOMA-IR) were measured. The authors observed a strong correlation between serum vaspin concentrations and VAT area when HOMA-IR was high [51]. Later on, Saboori et al. demonstrated that plasma vaspin level was higher in obese women than in normal weight women. In this study participants were matched by age and physical activity [60].

Finally, it is important to point out that a recent meta-analysis covering six studies with 1826 participants found significantly higher levels of serum vaspin in obese subjects [62]. The discrepancies in the relationship between vaspin serum concentrations and BMI or body fat values among the reported studies suggest that vaspin concentration may be dependent on several variables, such as gender, age, physical activity, hormonal metabolism (pubertal, period preceding menopause and post-menopausal women) or administered drugs [63]. 

Studies concerning the association between vaspin and obesity are not restricted to adults. Younger subjects have also been analyzed by other authors, although data are still scarce. Martos-Moreno et al. addressed a study in pre-pubertal Caucasian children and demonstrated that obese children had similar vaspin levels compared with lean controls [64]. By contrast, higher circulating visfatin levels in obese children and a positive correlation with BMI had been previously reported by other authors. Thus, Ko et al. found that elevated vaspin concentrations were related to obesity in pre-pubertal Korean children, after adjustment for sex, blood pressure, biochemical parameters, and other adipokines [55]. The authors suggested that the discrepancy may be partly explained by different population origins of subjects, or different definitions of obesity in children. In addition, the study reported by Ko et al. had a larger sample size and all children were the same age.

In several studies showing reductions in body weight, associated to different strategies, decreases in serum concentrations of vaspin have been found, reinforcing the idea that there is an association between obesity and this adipokine. Thus, Chang et al. reported decreased concentrations of serum vaspin after a modest weight loss in obese subjects, accompanied by improvements in parameters of insulin sensitivity [65]. The weight reduction program consisted of 12 weeks of individual intervention sessions designed to implement behavioral strategies related to eating and physical activity. Participants were instructed to reduce their daily energy intake by 500 kcal and were administered Orlistat. This decrease in vaspin concentration was concomitant with reductions in waist circumference, which is a useful index of central obesity. A limitation of this study was that abdominal fat distribution was not measured. Nevertheless, taking into account that modest weight loss was associated with the preferential loss of visceral rather than subcutaneous fat, the authors proposed that the decreased waist circumference may reflect the reduction in the accumulation of abdominal, particularly visceral fat. Thus, decreased vaspin concentrations may be associated with the reduction of visceral fat during the weight reduction program. Notably, strong correlations between changes in serum vaspin concentrations and changes in body weight, BMI, waist circumference, and hip circumference during the intervention were observed only in insulin resistant subjects. The authors proposed no explanation for this issue. Other authors have analyzed the effects induced in vaspin by body weight reduction associated to bariatric surgery. Handisurya et al. reported decreased levels of vaspin in the serum of morbidly obese subjects 12 months after laparoscopic Roux-enY gastric bypass (RYGB) surgery [50]. Similarly, Golpaie et al. demonstrated that restrictive bariatric surgery was accompanied by a significant decrease in serum vaspin concentrations [66]. 

### 3.2. Vaspin and Alterations in Glycemic Control

The evidence concerning the association between vaspin and glycemic control is clear [62]. As a matter of fact, a connection between vaspin and glycemic control was established since this adipokine was initially identified in a genetically obese and diabetic rat model [38]. As described below significant correlations between vaspin and several parameters related to glycemic control have been identified in subjects showing different metabolic features. Kloting et al. carried out a study in Caucasian subjects with BMI ranging from 21 to 54. Some of them showed normal insulin sensitivity, others impaired glucose tolerance, and finally others were diabetic. Interestingly, vaspin mRNA expression was not detectable in lean subjects with normal glucose tolerance, but can be induced by increased fat mass, decreased insulin sensitivity, an impaired glucose tolerance. Vaspin mRNA expression was more frequently observed in subjects with type 2 diabetes. According to Wada (2008) [67] the authors suggested that induction of vaspin mRNA expression in human adipose tissue could represent a compensatory mechanism associated with obesity, severe insulin resistance, and type 2 diabetes [42]. In another study, carried out in normal weight Korean women who showed no alterations in glycemic control, serum vaspin concentration significantly correlated with fasting insulin and HOMA-IR [10].

Significant correlations were also found in subjects showing metabolic syndrome. Tan et al. observed that serum vaspin concentration was significantly higher in men with metabolic syndrome than in men without the syndrome. By contrast, no differences were observed in women. Moreover, among men with diabetes, vaspin concentration was lower in subjects with a longer duration of diabetes or microvascular complications [52]. 

In the above studies, significant correlations were found between several metabolic parameters and serum vaspin concentrations. In addition, Goktas et al. [41] reported that mesenteric adipose tissue vaspin protein concentrations were positively correlated with HOMA-IR and blood HbA1c levels in morbidly obese patients. Additionally, diabetic subjects had about 3-fold higher mesenteric adipose tissue vaspin concentrations than non-diabetic and pre-diabetic subjects. In this study diabetic and pre-diabetic subjects had higher plasma vaspin concentrations than non-diabetic subjects. 

In line with these observations, in a study devoted to analyzing serum vaspin concentrations in newly diagnosed diabetic-obese and newly diagnosed diabetic-lean patients, and to compare them with those of non-diabetic obese or healthy subjects, the authors found that serum vaspin concentration was higher in diabetic-obese than in non-diabetic obese individuals. Since the authors included in the study only newly diagnosed diabetic patients, they concluded that, at the beginning of type 2 diabetes mellitus, vaspin might have a compensatory role [61]. The authors also declared that after diabetes treatment, or in cases of longer duration of this pathology, serum vaspin concentrations may be reduced.

An interesting study was reported by Li et al. Firstly, they observed higher serum vaspin concentrations in subjects with type 2 diabetes than in those showing impaired glucose tolerance or normal glucose tolerance [68]. When diabetic subjects were treated with intensive insulin therapy, by using insulin pumps, both postprandial and fasting blood glucose were almost normalized within 7 days, and euglycemia was maintained for 2 weeks. Along with an improvement in glucose metabolism, they observed a dramatic decrease in fasting plasma vaspin concentrations in these patients. The reduction in plasma vaspin levels was found to be associated with the amelioration of insulin sensitivity, shown by the changes in HOMA-IR. The authors concluded that the regulation of circulating vaspin might have been influenced by metabolic control and insulin resistance. Decreased circulating vaspin concentration could mediate the improvement of insulin resistance in the patients with type 2 diabetes. These results further confirm that vaspin may play an important role in type 2 diabetes and may be a part of the protective mechanisms aimed at reducing insulin resistance in humans [68]. Also, Kadoglou et al. (2011) observed in patients with type 2 diabetes that 6 months of treatment with metformin monotherapy and the combination of rosiglitazone and metformin decreased serum vaspin [69]. Finally, Jian et al. reported that low circulating vaspin level can be used as risk factors for the progression of type 2 diabetes, based on a 2-year follow-up study that included patients with type 2 diabetes and non-diabetic. The authors pointed out that further prospective observational studies were required to explain this issue [70].

Although the vast majority of the reported studies show increased serum vaspin concentrations in diabetic subjects or associations between serum vaspin and parameters related to glycemic control, such as HOMA-IR, there also studies in the literature showing different results. Thus, von Loeffelholz et al. did not find any association between serum vaspin and HbA1c in a cohort of non-diabetic humans with widely varying BMI (15.8–47.8 kg/m^2^) [45]. Moreover, vaspin did not show significantly different serum levels among subjects with isolated impaired fasting glycemia, isolated impaired glucose tolerance, normal glucose tolerant or type 2 diabetes, even after adjusting per for age, sex and BMI, in the study reported by Tonjes et al. [71].

Finally, human pregnancy often confers a state of insulin resistance and hyperinsulinemia that may predispose some women to gestational diabetes mellitus when their pancreatic function is not sufficient to overcome the diabetogenic environment of pregnancy. When comparing serum vaspin level, mRNA and protein levels of vaspin in adipose tissue obtained from women with gestational diabetes mellitus (GDM) with those from women with normal glucose tolerance, Mm et al. reported higher vaspin plasma levels and increased gene and protein expressions in subcutaneous adipose tissue in GDM women [72]. However, serum vaspin levels did not reflect the degrees of insulin resistance in GDM women. Unfortunately, the authors did not provide further information concerning the putative regulatory mechanism of vaspin in the occurrence of GDM [72].

### 3.3. Signaling Pathways Involved in the Metabolic Actions of Vaspin

Little is known concerning the mechanisms of action of vaspin. As a serpin family member, mechanism of action of vaspin is based on the stabilization of the protease-serpin complex.

Klöting et al. reported that both peripheral and central vaspin administration decreased food intake in obese *db/db* and lean C57BL/6 mice. According to these results they suggested that vaspin inhibited a protease that cleaves a putative anti-orexigenic factor. The effects of vaspin treatment on reduced food intake may therefore be an indirect result of an increase in the stability and efficacy of an anti-orexigenic factor that subsequently mediates vaspin effects to suppress food intake. The observation that food intake returned to normal values after 1 day without vaspin treatment suggested to the authors that the inhibition of proteinase activity by vaspin was limited by the time taken for elimination of the vaspin-protease complex from the plasma, which is approximately 1 day [42]. In addition, the administration of vaspin (1 μg/kg body weight) in the arcuate nucleus of the hypothalamus significantly decreased food intake in rats. This effect was due to the decrease in NPY gene expression and the increase in POMC gene expression [34].

With regard to the effects of vaspin on glycemic control, in a study carried out by Heiker et al. the authors demonstrated that human kallikrein 7 (hk7) is the target protease of vaspin. In this study, interactions between vaspin and hk7 in human plasma were observed. Moreover, human insulin was identified as hk7 substrate, suggesting that vaspin anti-diabetic effects in vivo are due to an insulin stabilizing ability through the reduction of hk7 induced insulin degradation (instead of increasing insulin sensitivity) [73].

Vaspin may inhibit inflammatory processes by reducing inflammatory adipokines and this effect may be beneficial for improvement in insulin resistance. Since pioglitazone treatment up-regulates vaspin mRNA expression in rodents, vaspin may be one of the effector molecules under the control of peroxisome proliferator-activated receptor γ (PPARγ) [67].

## 4. Cardiotrophin-1

Cardiotrophin-1 (CT-1) is a protein isolated in 1995 from the supernatant of mouse embryonic corpuscles. The name CT-1 comes from its ability to induce a hypertrophic response in neonatal cardiac myocytes as judged by myocyte enlargement, organization of myosin light chain into sarcomeric units, and atrial natriuretic peptide (ANP) secretion [74]. The coding region of human CT-1 gene is located on chromosomal region 16p11.1–16p11.2 [75]. Moreover, it has been described that the coding region of exon 1, 2 and 3 shows a homology of 96%, 84% and 81% respectively between human and mouse [76]. CT-1 protein consists of 203 amino acids and has a molecular weight of 21.5 kDa [77] also presenting a sequence well conserved between species. Thus, the protein homology between human and mouse is 78%, and 80% between human and rat. The rat amino acid sequence is 94% identical to that of mouse CT-1 [76]. 

The initial studies with CT-1 showed that its structure was very similar to the interleukin-6 (IL)-6 family of cytokines, and was considered as a member of this family [74]. The IL-6 family includes other cytokines such as IL-11, IL-30, ciliary neurotrophic factor (CNTF), cardiotrophin-like cytokine (CLC), also known as novel neurotopin-1 (NNT-1) or B cell stimulating factor 3 (BSF3), neuropoietin (NP), leukemia inhibitory factor (LIF), oncostatin M (OSM) and IL-31 [78]. All the cytokines of this family induce their physiological actions by activating the gp130 receptor, which causes some of them to share certain features; however, the different distribution of this receptor and the fact that each cytokine binds to a more specific receptor, make that these cytokines are not considered as redundant in many of their functions [79].

CT-1 is expressed in many tissues, although the specific physiological role in most of them remains to be elucidated. In adult humans, CT-1 is highly expressed in heart, skeletal muscle, liver, lung and kidney. Lower levels of CT-1 expression are also seen in testis and brain [75]. Interestingly, adipose tissue has been also identified as a source of CT-1, acting as an adipokine [80,81]. Therefore, CT-1 could act not only in a paracrinic manner, but also as an endocrine factor involved in the regulation of several physiological/pathophysiological functions [82].

Interestingly, a recent study has revealed that a 24-h circadian rhythm of CT-1 plasma levels was observed in normal-weight subjects, which was altered in overweight patients. Chronodisruption has a great influence on metabolic disturbances and vice versa [83]. Although the exact mechanisms linking metabolic syndrome with chronodisruption are still unknown, most hypotheses point to an internal desynchronization of circadian rhythms involved in metabolism [84]. It is important to highlight that several adipokines secreted by adipose exhibit profound day/night circadian rhythms, and accumulating evidence links disruption of these rhythms to the development of clinical metabolic disturbances [85]. 

### 4.1. Cardiotrophin-1 and Obesity

In recent years, a large number of investigations have focused on analyzing the ability of CT-1 to regulate body weight and intermediate metabolism [80,86,87]. In this sense, it has been described that CT-1 deficiency brings on late-onset adult obesity in mice, accompanied by insulin resistance and hypercholesterolemia [86]. It is important to highlight that *CT-1*^−/−^ mice, by contrast with other deficiencies in gp130 ligands, develop obesity despite reduced food intake probably as a consequence of decreased energy expenditure. Hence, CT-1 deficient mice represent a hypophagic model of obesity [86]. Moreover, oxygen consumption rate (*VO*_2_) circadian rhythmicity was disrupted in aged obese CT-1-deficient mice as compared to wild-type mice [83]. Interestingly, the circadian rhythm exhibited for key clock genes (*Clock*, *Bmal1*, and *Per2*) in adipose tissue was altered in *CT-1*^−/−^ mice, which showed a lower percentage of the rhythm or lower amplitude, especially for *Bmal1* and *Clock*. These data suggest that CT-1 could be important in the regulation of metabolic circadian rhythms and adipose core clock genes in mice.

However, there is still some controversy regarding the regulation of CT-1 levels during obesity both in rodents and humans. Indeed, studies performed in mice have shown that gene expression levels of CT-1 were down-regulated in white adipose tissue from diet-induced obese mice [88,89]. Moreover, plasma levels of this cytokine are elevated in patients with obesity and metabolic syndrome, suggesting that CT-1 could be considered as a marker and a link between adipose tissue, insulin resistance and cardiovascular diseases [80,81,82,83,84,85,86,87,88,89,90,91]. Contrariwise, another study stated that overweight and obese subjects had significantly lower CT-1 levels than those with normal weight [92]. Another investigation, conducted in obese adolescents, did not find any differences in circulating levels of CT-1 compared to normal-weight subjects of the same age [93]. Similarly, a recent study by López-Yoldi et al. [83] did not observe any significant changes in plasma CT-1 from normal weight subjects when compared to overweight/obese individuals, suggesting that other factors associated to obesity, and not the increased adiposity directly, could be determining the levels of this cytokine. So, the role of CT-1 in obesity in humans remains to be further elucidated.

### 4.2. Cardiotrophin-1 and Alterations in Glycemic Control

Concerning to the effects of CT-1 on glucose metabolism, a study performed by Moreno-Aliaga et al. (2011) [86] revealed CT-1 hypoglycemic properties, which were mediated by insulin-dependent and insulin-independent mechanisms. Indeed, the glucose-lowering properties of CT-1 were also observed in mice with streptozotocin-induced insulin deficiency, thus evidencing an insulin-independent effect of CT-1 on glucose metabolism. To further support the role of CT-1 on glucose homeostasis, this study showed the ability of this adipocytokine to promote insulin-stimulated glucose uptake in L6E9 myotubes and in 3T3-L1 adipocytes [86].

### 4.3. CT-1 Receptor and Signaling Pathways Involved in the Metabolic Actions of CT-1

All members of the IL-6 family share gp130 as a signal-transducing receptor component. Thus, these cytokines induce their physiological actions by activating the gp130 receptor and require interaction with two gp130 or one gp130 and another related signal transducing receptor subunit [94]. CT-1 requires gp130 and LIF receptor (LIFR) heterodimerization to induce signal transduction. Although CT-1 can mimic LIF in binding and activation of the gp130/LIFR complex, it has been reported to recruit an as yet uncharacterized α receptor (CT-1Rα) [95], which confers CT-1 high potency trophic signaling for motor neurons [75,77,95]. However, this additional membrane component is apparently not required for CT-1 effects on other cells [96]. Intracellular signaling initiated after phosphorylation of gp130 basically involves several families of intermediary molecules, including the PI3K/AKT system, the Janus kinase/transducer and activator of transcription (JAK/STAT) signal system, the mitogen activated protein kinase/extracellular signal-regulated kinase (MAPK/ERK) system and the nuclear factor κB (NF-κB) system [82]. 

**Figure 2:**
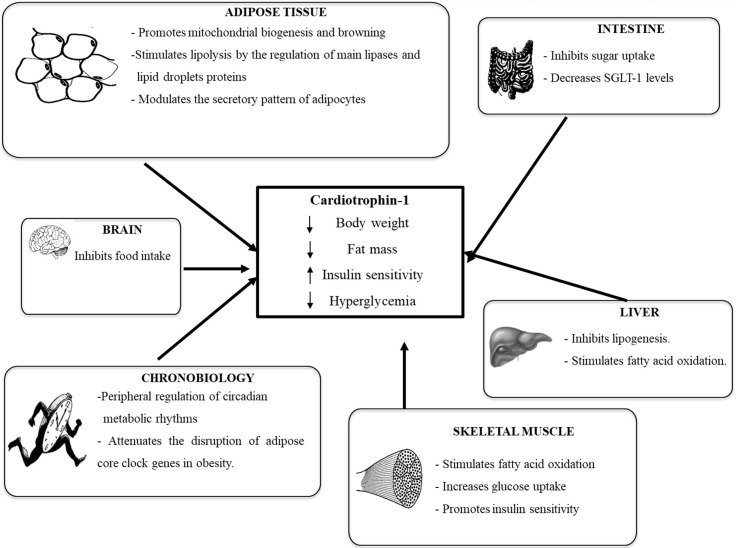
Schematic representation of the metabolic actions of cardiotrophin-1. This adipokine promotes body weight and fat mass losses, reduces hyperglycemia and increases insulin sensitivity by coordinately acting on key metabolic tissues (hypothalamus, adipose tissue, liver, intestine and muscle). SGLT-1: sodium-dependent glucose transporter 1.

Figure 2 summarizes the main metabolic actions underlying the anti-obesity and anti-diabetic properties of CT-1. In this context, activation of hypothalamic pathways involved in hypophagia, including signal transducer and activator of transcription 3 (STAT-3) and S6 ribosomal protein (S6) has been involved in the anorexigenic actions of CT-1 [86]. 

As stated before, white adipose tissue produces CT-1, but it is also a target organ for this protein. CT-1 activates major signaling pathways involved in the control of metabolism in this tissue [80,97]. No studies are available in human white adipose tissue biopsies, but studies performed in mice revealed that chronic CT-1 administration induced a dramatic remodeling of white adipose tissue, characterized by a reduction of adipocyte size accompanied by the down-regulation of lipogenic genes and up-regulation of genes involved in lipid catabolism [86]. Furthermore, CT-1 promotes mitochondrial biogenesis and adipose tissue browning [86].

The lipolytic properties of CT-1 in adipose tissue are mainly mediated through the protein-kinase A (PKA) pathway. Indeed, CT-1 treatment promoted an increase in cAMP levels in cultured 3T3-L1 adipocytes, thus activating PKA. These facts result in the PKA-mediated phosphorylation of perilipin and hormone sensitive lipase (HSL) at Ser660, promoting lipolysis [98]. In line with the results observed in cultured adipocytes, acute CT-1 treatment is also responsible for the PKA-mediated phosphorylation of perilipin and HSL at Ser660 and Ser563 in *ob/ob* mice [98].

In addition, CT-1 also modulates the adipokine secretory pattern of adipocytes. Thus, CT-1 treatment increases apelin gene expression and secretion, while downregulates the production of leptin, resistin and visfatin in 3T3-L1 adipocytes [81]. Furthermore, acute administration of CT-1 to diet-induced obese mice downregulated leptin and resistin gene expression, without significantly modifying apelin mRNA levels in adipose tissue [81]. It is important to highlight that during the last decade, white adipose tissue has been established as an important endocrine organ with a key relevance in the regulation of food intake, energy expenditure and glucose and lipid homeostasis [99]. Thus, the ability of CT-1 to modulate the production of adipokines, in vitro and in vivo, suggests that the regulation of the secretory function of adipocytes could be also involved in the metabolic actions of this cytokine.

As far as glycemic control is concerned, another study has demonstrated that in vivo administration of CT-1 inhibits intestinal sugar uptake after acute and chronic treatments. Indeed, studies in Caco-2 cells revealed that CT-1 decreased sugar absorption by reducing the protein levels of the sodium-dependent glucose transporter 1 (SGLT-1) [100]. The inhibition of intestinal sugar uptake could also account for the hypoglycemic and anti-obesity properties of CT-1. In this sense, agents that inhibit glucose co-transporters (SGLT inhibitors) that mediate intestinal glucose absorption or increase glucose excretion, could help to control hyperglycemia through an insulin-independent mechanism, introducing a new concept to the diabetes treatment [101].

Another pathway that plays an important role mediating the metabolic properties of CT-1 is PI3K/AKT pathway. Obese CT-1-deficient mice exhibited reduced insulin-stimulated AKT phosphorylation in both skeletal muscle and white adipose tissue. On the contrary, a stimulation of insulin-stimulated AKT phosphorylation was observed in muscle of mice treated with rCT-1 (recombinant cardiotrophin-1) [86] in parallel with a significant reduction in glycaemia and insulinemia. In this context, it has been demonstrated that AKT is a major mediator in the stimulatory effect of rCT-1 in glucose uptake by cultured myocytes and adipocytes [86]. Moreover, the PI3K/AKT pathway seems to be also mediating the stimulatory effects of rCT-1 on apelin gene expression and secretion in cultured 3T3-L1 adipocytes, since pre-treatment with the PI3K inhibitor LY294002 partially reversed this effect [81]. AMPK activation is also involved in the metabolic effects of CT-1 in liver and skeletal muscle, where it increases fatty acid oxidation, [86,87]. Interestingly, the AMPK signaling pathway could also mediate the inhibitory effects of CT-1 on α-Methyl-d-glucoside uptake observed in Caco-2 cells. Thus, when cells were pre-treated in the presence of the JAK/STAT inhibitor (AG490) and with the AMPK activator (AICAR), the inhibitory effects of CT-1 on sugar uptake and SGLT-1 levels were abrogated [100].

## 5. TWEAK

Tumor necrosis factor-like Weak Inducer of Apoptosis (TWEAK) was discovered in 1997 [102]. TWEAK is also known as Tumor necrosis factor (TNF) ligand superfamily member 12, TNFSF12 [103], and it, together with its receptor, FGF-inducible molecule-14 (Fn14, also known as TNFRSF12A), is a member of the TNF/TNFR superfamily, which regulates several tissue responses, sometimes apparently conflicting [104]. Recently, TWEAK has been a focus of attention in the research community as a potential regulator of the low-grade chronic inflammation characteristic of obesity (extensively reviewed in Burkly [105]).

*TWEAK* gene is located at chromosomal position 17p13.1, encoding initially for a 249 amino acid transmembrane protein [102]. Then this transmembrane protein is processed by cleavage by a furin endoprotease, leading to the release of a mature soluble form of TWEAK of 18 kDa [102]. Cells can co-express both plasma membrane-anchored and soluble TWEAK [106]. However, membrane-anchored TWEAK is rarely detectable as a consequence of efficient cleavage by furin [107]. TWEAK is expressed in a wide variety of different tissues and cells, including tumor cell lines, intestine, pancreas, lung, brain, ovary, skeletal muscle, vasculature, kidney, liver and adipose tissue [108,109,110,111,112,113,114,115]. By contrast, Fn14 expression in healthy tissues is usually very low. However, under pathological conditions, Fn14 expression is rapidly induced in several tissues such as liver [116], heart [117], colon [118], skeletal muscle [119], as well as during atherosclerosis [120] and kidney injury [121]. Several studies have evidenced expression of TWEAK and Fn14 in visceral adipose tissue and also in subcutaneous adipose tissue [111,122]. Interestingly, some human studies have deciphered TWEAK as a component of the network that contributes to the inflammatory imbalance observed in obesity [111,122,123,124].

### 5.1. TWEAK, Obesity and Alterations in Glycemic Control

Some studies have focused on the potential relationship between TWEAK levels and obesity, but overall on understanding the relevance of TWEAK in morbid obesity. Nevertheless, the data reported are sometimes controversial. In morbidly obese patients TWEAK and Fn14 expression were increased in adipose tissue compared to their lean counterparts [124]. Obesity is associated with a chronic low-grade inflammatory state, specifically in adipose tissue. Under these circumstances, TWEAK expression has been detected mainly in immune cells [125], whereas Fn14 expression has been mainly detected in pre-adipocytes and adipocytes [124,126,127] or endothelial cells [112].

Interestingly, TWEAK knock-out mice fed on a high fat diet showed more susceptibility to develop adult obesity than the wild-type mice [128]. However, these TWEAK knock-out mice were protected from dyslipidemia and ectopic fat deposition, exhibiting enhanced whole body insulin action and glucose tolerance, as well as improved insulin signaling in liver and muscle [128]. Expression levels of both TWEAK and Fn14 were higher in gonadal fat of wild-type mice fed on a high fat diet [128]. By contrast, Maymó-Masip et al. [124] reported that TWEAK circulating levels were highly variable in extremely obese subjects, although globally they were lower than in their lean counterparts. Moreover, lower TWEAK levels have been associated with higher levels of glucose, HOMA-IR index and visceral obesity [129,130] and higher TWEAK levels have been positively related to triglycerides [131]. Finally, other studies reported no association between TWEAK and obesity [132]. On the other hand, in a situation of a huge adipose tissue loss, such as occurs in the lipodystrophy associated to the HIV infection, TWEAK levels were lower compared with their controls counterparts [133]. Increased levels of TWEAK in skeletal muscle favors the development of visceral obesity, accompanied with insulin resistance, and metabolic dysfunction, suggesting that TWEAK inhibition could be a potential approach to prevent weight gain and type 2 diabetes [134].

Taken together, these observations suggest that TWEAK levels are not directly related to adiposity, and that other tissues or metabolic factors are playing a critical role in TWEAK levels regulation. Moreover, the role of TWEAK in obesity and in the risk of metabolic complications seem to be tissue-dependent. Consequently, further studies with larger cohorts are needed to better characterize TWEAK/Fn14 role in obesity-associated metabolic disturbances.

### 5.2. TWEAK Aeceptors and Signaling Pathways Involved in the Metabolic Actions of TWEAK

Fn14 was identified as the specific TWEAK receptor [135]. The *Fn14* gene is located at the 16 chromosome at position p13.3 [136], encoding a transmembrane protein of 14 kDa that is processed into a mature form of 102 amino acids [135]. Fn14 interacts with TWEAK at the extracellular domain [137] and transduces the TWEAK signaling by a tumor necrosis factor receptor-associated factor (TRAF) binding site interacting with the TRAF family proteins [137]. Moreover, in monocytes/macrophages, it has been described a scavenger receptor for TWEAK, CD163 [138,139] which avoids TWEAK actions possibly by sequestering this adipokine from the environment. Furthermore, it has been proposed the existence of other signaling receptors different than Fn14, because in vitro culture cells response to TWEAK in an Fn14 independent manner [140]. Nevertheless, the significance of CD163 and other potential signaling receptors needs to be confirmed with more studies in order to discern the relevance of them in the TWEAK signaling.

Treatment with TWEAK triggers NF-κB activation in several cell types [141,142], and this activation has also been connected to an increased secretion of proinflammatory cytokines [143,144,145,146]. NF-κB complexes are dimers with DNA-binding capabilities and transactivating domains. NF-κB is regulated by their subcellular localization. Thus, NF-κB is sequestered in the cytoplasm by the IκB proteins (inhibitor of κB) in the inactive state. Upon treatment with high concentrations of TWEAK, Fn14 recruits TRAF proteins and IκB is degraded allowing nuclear translocation of the dimmers [141]. Once inside the nucleus, NF-κB activates the expression of over 400 target genes, promoting a weak cell death by apoptosis. However, at physiological concentrations, TWEAK is unable to activate canonical NF-κB, but strongly activates the non-canonical NF-κB [147,148]. In adipocytes, TWEAK treatment promotes a low inflammatory grade, through activation of NF-κB pathways, inducing the expression of the pro-inflammatory cytokines MCP-1 and IL-6 [122,127]. Moreover, TWEAK treatment is also able to modulate other intracellular signaling through the activation of two of the MAPK pathways: ERK1/2 [122] and p38 [149] but not JNK [122] in adipocytes. Finally, insulin signaling is a key regulator of adipocyte metabolism and it is also modulated by TWEAK bennett [128].

In Tweak knock-out mice, under a high fat diet, in vivo insulin signaling (AKT phosphorylation) was enhanced as compared with control mice counterparts [128]. AMP-activated protein kinase (AMPK) activity is also regulated by TWEAK. Indeed, muscle-specific overexpression of TWEAK inhibited AMPK and upstream kinase LKB1, without affecting the Akt pathway. The activation of GSK3β, which directly interacts and inhibits AMPK activity, was found to be increased in the skeletal muscle of TWEAK-transgenic mice. In this model, TWEAK also represses the levels of Krüppel-like factor 15 (KLF15) and myocyte enhancer factor 2 (MEF2), and peroxisome proliferator-activated receptor-γ coactivator-1a (PGC-1α), which are required for GLUT4 expression [134].

## 6. NOV/CCN3

NOV/CCN3 (nephroblastoma overexpressed) protein in humans is encoded by the *nov* gene. This protein is a member of an emerging family of six regulatory proteins Cyr61/CCN1 (cystein rich protein 61), Ctgf/CCN2 (connective tissue growth factor), Nov/CCN3, ELM-1/WISP-1/CCN4 (WNT1-inducible-signaling pathway protein 1), rCop-1/WISP-2/CTGF-L/CCN5 (WNT1-inducible-signaling pathway protein 2), and WISP-3/CCN6 (WNT1-inducible-signaling pathway protein 3) [150,151].

The *nov* gene is detected in various human tissues, such as adrenal cortex, kidney, muscle, heart, adipose tissue and central nervous system among others, in macrophages and in biological fluids, including amniotic and cerebrospinal fluids and plasma [152,153,154,155,156]. It was first characterized as an integration site for the myeloblastosis associated virus [157], which promotes kidney tumors resembling nephroblastoma and Wilms tumor perbal [158]. In both human and animal tumors, its gene expression was found to be altered, either up-regulated or down-regulated [159,160,161,162,163].

Nowadays, it is known that, in addition to its relation to cancer, NOV/CCN3 plays an important role in organogenesis, inflammation or fibrosis [164,165]. All these functions are mediated by its interaction with integrins and by the Notch pathway, which regulates cell-fate during development and maintains adult homeostasis tissue [164].

### 6.1. NOV/CCN3 and Obesity

Martinerie et al. (2016) studied the effects of NOV/CCN3 in 3T3-L1 pre-adipocytes and primary pre-adipocytes. In 3T3-L1 pre-adipocytes NOV seems to decrease adipogenesis, since when cells were transfected by a specific siRNA against NOV, that is this adipokine was blunted, the expression of sterol regulatory element-binding protein 1c (SREBP-1c), peroxisome proliferator-activated receptor γ (PPAR-γ), and CCAAT-enhancer-binding protein α (C/EBP-α) increased. In addition, pre-adipocytes obtained from NOV^−/−^ mice adipose tissue differentiated better than those from control mice, as it was observed by the increase in SREBP-1c, PPAR-γ, and C/EBP-α expressions [166].

Moreover, Pakradouni et al. [155] compared plasma concentration and gene expression of this protein in C57/Bl6 mice fed a high-fat or a standard diet for 12 weeks. They observed that obese mice (those fed the high-fat diet) showed higher plasma levels than lean mice. Moreover, nov mRNA level in the visceral adipose tissue was also increased in high-fat fed mice compared with standard diet-fed mice. More recently, Martinerie et al. [166] compared NOV/CCN3 (*NOV*^−/−^) knock-out mice and wild-type mice fed for 16 weeks either a high-fat or a standard diet. When the animals were fed the standard diet no differences in weight gain or fat mass were observed. By contrast, *NOV*^−/−^ mice, but not wild-type mice, were protected against diet-induced obesity and adipose tissue inflammation. This different behavior of knock-out and wild type mice occurred without changes in food intake or differences in lipid gut absorption. However, there was slightly enhanced energy expenditure during the daylight period that was associated with a potential induction of thermogenesis, as it was concluded by the increase in uncoupling protein-1 (UCP1) and proliferator-activated receptor gamma coactivator 1 alpha (PGC1-α). 

In humans, Pakradouni et al. [155] studied plasma concentration of NOV/CCN3 in adults showing hyperlipidemia with or without lipid-lowering therapy. They showed, for the first time, a strong correlation between plasma NOV/CCN3 concentration and BMI or fat mass. Interestingly, they found gender differences, thus women displayed higher level of circulating NOV/CCN3 than men. Such effect could be attributed to the higher proportion of fat content in women and/or to other mechanisms, such as hormone status. Also, plasma triglycerides, but not cholesterol, were weakly associated with NOV/CCN3 concentration. When patients lost weight after gastric bypass circulating NOV/CCN3 concentrations decreased. 

### 6.2. NOV/CCN3 and Alterations in Glycemic Control

Several evidences suggesting the role of NOV/CCN3 on glycemic control have been reported. Martinerie et al. (2016) observed that in 3T3-L1 adipocytes transfected with siRNAs to silence NOV/CCN3, the decrease in the expression this gene was accompanied by an increase in insulin-stimulated p-Akt and p-Erk, compared with the control cells [166]. 

On the other hand, Paradis et al. (2013) demonstrated that Forkhead box O1 protein (FoxO1), a transcription factor that regulates genes involved in diabetes development, regulated *ccn3* gene expression in pancreatic β-cells. Thus, over-expression of FoxO1 increased *ccn3* mRNA levels, whereas the inhibition of the transcription factor caused the opposite effect [167]. In good accordance with this in vitro observation, Martinerie et al. observed that FoxO1 activation increased *ccn3* in transgenic mice. On the other hand, NOV/CCN3 inhibited β-cell proliferation and thus impaired β-cell insulin secretion [166]. 

These authors also reported that NOV/CCN3 contributed to the development of obesity-induced insulin resistance, based on their studies in knock-out mice. Thus, in the experiment previously described in this review, the authors observed that *NOV*^−/−^ mice were protected against diet-induced glucose intolerance and insulin resistance [166]. Finally, Shimoyama et al. [168] demonstrated that NOV/CCN3 expression was reduced in the aorta from mice that developed diabetes after streptozotocin injection. By contrast, its expression was increased by insulin treatment. 

With regard to human beings, in the study reported by Pakradouni et al. [155], carried out in adults showing hyperlipidemia with or without lipid-lowering therapy, HbA1c which is an important blood marker that gives a good indication of long term glucose levels, was positively correlated with circulating NOV/CCN3 levels. However, no correlation was found between plasma NOV/CCN3 concentration and blood glucose.

## 7. Concluding Remarks

As far as obesity is concerned, it has been reported that this pathology reduces serum omentin-1 concentration and adipose tissue secretion in adults and adolescents. In the case of children, the reduced number of reported studies and the discrepancies indicate that further studies are needed. Conversely, the strategies used to induce a reduction in body weight, such as dietary energy restriction and physical activity, are effective in correcting the alterations induced in omentin-1 serum concentration induced by overweight and obesity. Animal studies suggest that omentin could be involved in the regulation of appetite due to the neuromodulatory role of this adipokine on peptidergic and aminergic signaling. Moreover, omentin increases insulin-stimulated glucose transport by increasing protein kinase b (Akt) phosphorylation, as well as the activity of IRS.

Higher levels of vaspin have been found in obese subjects when compared with normal weight subjects. On the other hand, in several studies, showing reductions in body weight associated to different strategies, decreases in serum concentrations of vaspin have been found, reinforcing the idea that an association between obesity and this adipokine exists. Vaspin reduces food intake and may inhibit inflammatory processes by reducing inflammatory adipokines and this effect may be beneficial for improvement in insulin resistance.

In recent years, a wide number of investigations have focused on analyzing the ability of cardiotrophin-1 to regulate body weight and intermediate metabolism. The vast majority of the studies addressed so far have been conducted in mice. In this animal model, cardiotrophin-1 induces a dramatic remodeling of white adipose tissue, characterized by a reduction in adipocyte size, accompanied by the down-regulation of lipogenic genes and the up-regulation of genes involved in lipid catabolism. Furthermore, this adipokine promotes mitochondrial biogenesis and adipose tissue browning.

Recently, TWEAK has focused attention of the research community as a potential regulator of the low-grade chronic inflammation characteristic of obesity. TWEAK levels are not directly related to adiposity, and other tissues or metabolic factors are playing a critical role in TWEAK levels regulation. Moreover, the role of TWEAK in obesity and risk of metabolic complications seem to be tissue-dependent. Consequently, further studies with larger cohorts are needed to better characterize TWEAK/Fn14 role in obesity-associated metabolic pathologies.

Finally, a strong correlation has been observed between NOV/CC3 plasma concentration and body max index or fat mass. When patients lose weight, after gastric bypass, circulating NOV/CCN3 concentration decreases.

**Figure 3:**
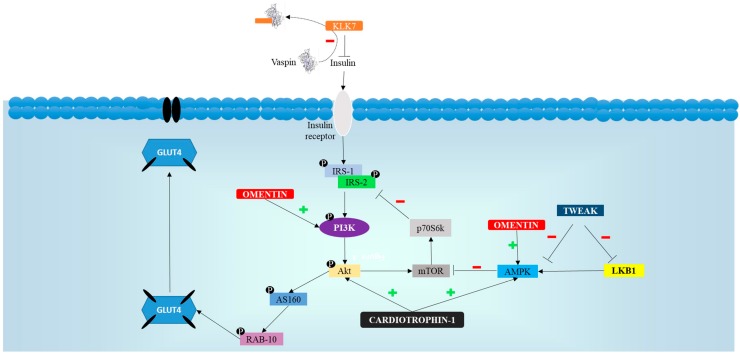
Schematic representation of the effects of omentin, vaspin, cardiotrophin-1, TWEAK and NOV/CCN3 on insulin signaling cascade that explain positive effects of these adipokines on glycemic control. Akt: protein kinase b; AMPK: AMP activated protein-kinase; AS160: Akt substrate of 160 kDa; GLUT: glucose transporter; IRS: insulin receptor substrate; LKB1: liver kinase B1; mTOR: mammalian target of rapamicin; P: inorganic phosphorous; PI3K: phosphatidylinositol 3-kinase; RAB-10: Ras-related protein RAB-10.

Figure 3 summarizes the effects of omentin, vaspin, cardiotrophin-1, TWEAK and NOV/CCN3 on insulin signaling cascade.

With regard to alterations in glycemic control, several authors have documented lower levels of serum omentin-1 in nascent metabolic syndrome, type 2 diabetes or even type 1 diabetes. It has been proposed that omentin-1 improves insulin sensitivity. As far as vaspin is concerned, the reported results suggest that vaspin may play an important role in type 2 diabetes, and may be a part of the protective mechanisms aimed at reducing insulin resistance in humans. Data concerning the involvement of cardiotrophin-1 on glucose homeostasis have been obtained in mice. In this animal species, this adipokine reduces intestinal glucose absorption and improves insulin sensibility.

In addition, lower TWEAK levels have been associated with higher levels of glucose, HOMA-IR index and visceral obesity. Finally, there is just a study addressing the effects of NOV/CCN3 on glycemic control in humans where HbA1c, which is an important blood marker that gives a good indication of long term glucose levels, shows positive correlation with circulating NOV/CCN3 levels, but no correlation with blood glucose.

**Figure 4:**
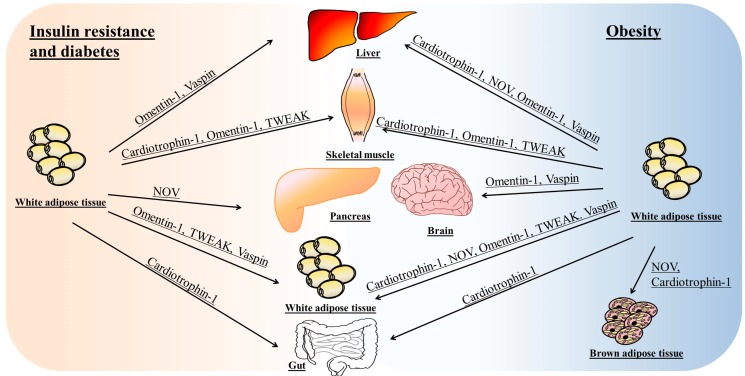
Schematic representation of the main tissues and organs involved in the effects of omentin, vaspin, cardiotrophin-1, TWEAK and NOV/CCN3 on insulin resistance and obesity. On the left side of the figure, target tissues and organs involved in the effects of adipokines on insulin resistance. On the right side of the figure, target tissues and organs involved in the anti-obesity action of adipokines.

Figure 4 summarizes the main tissues and organs involved in the effects of omentin, vaspin, cardiotrophin-1, TWEAK and NOV/CCN3 on insulin resistance or obesity.
